# Evaluation of a perinatal palliative care program by SWOT analysis

**DOI:** 10.1038/s41390-024-03366-2

**Published:** 2024-09-12

**Authors:** Francesca Catapano, Giuseppe Ramacieri, Giacomo Sperti, Luigi Tommaso Corvaglia, Chiara Locatelli

**Affiliations:** 1 Department of Medical and Surgical Sciences (DIMEC), University of Bologna, Via Massarenti 9, 40138 Bologna, Italy; 2 School of Specialization in Child Neuropsychiatric, University of Bologna, Via Massarenti 9, 40138 Bologna, Italy; 3 Perinatal comfort care and assistance to the newborn with congenital malformations Unit, Department of Neonatology, IRCCS Azienda Ospedaliero-Universitaria di Bologna, Bologna, Italy; 4 Neonatology Unit, IRCCS Azienda Ospedaliero-Universitaria di Bologna, Bologna, Italy

## Abstract

**Background:**

Perinatal Palliative Care (PPC) is individualized medical-nursing care aimed at improving the quality of life of newborns with life-limiting conditions and to support their families. This study draws on the analysis of the experience gained over ten years by a service of PPC called the “Percorso Giacomo” (PG).

**Methods:**

We employed a SWOT analysis to identify the strengths, weaknesses, opportunities, and threats of the PG through a systematic retrospective review of 48 cases followed by the program over the course of 10 years, 21 unsolicited parents’ narrative and 27 experts’ point of view.

**Results:**

Main strengths of the program were communication and parents’ involvement in shared decision-making. Main weaknesses included lack of knowledge of the presence and the role of the PG and lack of resources. For opportunities, the PG proved to be an innovative choice for pregnancies with a fetal life-limiting diagnosis, however threats were identified such as lack of knowledge of PPC and delayed referrals.

**Conclusion:**

The analysis by SWOT method of the 10-year experience of the PG allowed the identification of limitations and areas of improvement, however demonstrated that the PG provided beneficial services to women faced with fetal life-limiting diagnoses.

**Impact:**

- Perinatal Palliative Care (PPC) practice and literature on this subject is still limited.
- This study offers features of the 10-year experience of the Percorso Giacomo (PG), a service of PPC, through a SWOT analysis.
- By identifying strengths, weaknesses, opportunities and threats of the PG, the study shows limitations and areas of improvement but also benefits of a PPC service to women with fetal diagnosis of life-limiting condition and may allow replication in other institutions.

## Introduction

First introduced in the 1960s, the concept of palliative care was developed in England by Dame Cicely Saunders. She was a social worker, a nurse and a physician, encompassing the three essential figures of palliative care and, while caring for patients at the last stage of their lives, focused above all on the alleviation of pain in all its forms – physical, emotional, and spiritual.^1^

Gradually, palliative care moved from a purely adult-related field to a pediatric one, and eventually to a perinatal one, recognizing that pain and discomfort can affect newborns, regardless of gestational age.^2–4^ Perinatal Palliative Care (PPC) addresses the entire perinatal journey, and it is offered in the case of a fetal or neonatal diagnosis of life-limiting (LLC) and life-threatening (LTC) conditions as a plan to achieve the comfort of the newborn and to support the mother and the family from a medical, emotional, psychological and spiritual points of view.^5^ Recent biotechnological advances have made it possible to detect the health status of the developing fetus at an increasingly early stage. Although the absolute number of problems detected has not increased, the number of prenatal investigations has increased exponentially, leading to a growing number of families facing a diagnosis of congenital malformations including serious genetic conditions before the birth.^6,7^ After such serious diagnostic communication, pregnancy becomes a time of apprehension and uncertainty.^8,9^ Thus, it is essential to give parents honest and non-directive information to guide them toward an informed choice regarding the pregnancy management and the clinical care of the infant.^10,11^

The importance of PPC has been recognized by the American College of Obstetrics and Gynecologists (ACOG) that recommends PPC as one of the options, along with termination of pregnancy and neonatal intensive care, to be offered to families facing a fetal diagnosis of LLC.^12^

Although palliative care has been established as a discipline to support adult and pediatric patients, this is not true for PPC. Currently, there are no international standardized protocols for PPC,^13^ and this discipline is not included in everyday clinical practice in the United States, and elsewhere, including Italy.^14–16^ However, given the scientific evidence of the benefit derived from a PPC approach^17–21^ and the recognition of its importance by major perinatal organizations, namely the American Academy of Pediatrics (AAP)^22^ and the Italian Society of Neonatology (SIN),^23^ some services of PPC have been implemented in Italian institutions.^24^

This is the case of “Percorso Giacomo” (PG) or “Giacomo’s Pathway” a program of PPC created in 2013 at Sant’Orsola Hospital (SOH) in Bologna, Italy^24^ following guidelines established by the Neonatal Comfort Care Program (NCCP) at the Columbia University Irving Medical Center (CUIMC) in New York, NY.^25^

## Objective

The aim of this research is to evaluate the 10-year experience of the PG to assess the features (strengths, weaknesses, opportunities, and threats) that may allow its replication in other institutions.

## Materials and methods

This research was approved by the ethics committee of the Sant’Orsola Hospital in Bologna, Italy.

A SWOT analysis was conducted to identify external (opportunities, threats) and internal (strengths and weaknesses) components of the PG program based on a systematic retrospective review of patient medical records, parents’ narratives and experts’ point of view.

The investigation was divided in 2 phases.

### First phase

Medical records of pregnant women and of their newborns followed by the PG from January 1^st^ 2013 to March 31^st^ 2024 were inspected. This is a selected population of women who elected to continue their pregnancy in the settings of a fetal LLC and to establish a postnatal plan of comfort care in case of livebirth.

Data collected included: fetal diagnosis and gestational age at the time of first encounter with the PG team and written narratives from each encounter’s debriefing, both prenatally and postnatally. A post-encounter debriefing is a critical component of the PG’s each case management – whether the pregnancy outcome is fetal demise, stillbirth, livebirth followed by neonatal death – and is aimed at pointing out what went well and potential areas for improvement. We also collected written unsolicited feedback from families. Through content analysis we obtained 16 attributes.

### Second phase

The SWOT (Strengths, Weaknesses, Opportunities and Threats) analysis^26,27^ was obtained from the results of the content analysis. The SWOT categories were subsequently assessed through an anonymous questionnaire designed on a Qualtrics platform. The survey included participants’ demographic information and the SWOT categories to be assessed with quantitative Likert agreement/disagreement scale (1= strongly disagree; 2= disagree; 3= neutral; 4= agree; and 5= strongly agree). Participants, including professionals and parents were selected through purposive sampling. Inclusion criteria for health care providers were participation at least once to the care of families followed by the PG, and experience in treating infants with LLC. Inclusion criteria for parents were perinatal loss at least 5 years prior to the study period and Italian speaking. Participants received the questionnaire via email, after they had consented to participate. The results were analyzed using descriptive statistics.

## Results

Forty-eight women with fetal LLC were followed by the PG team and the first encounter for 87% occurred in the third trimester. Fetal diagnoses included Anencephaly (*N* = 7), Renal dysplasia (*N* = 8), Trisomy 18 (*N* = 9), Trisomy 21 complicated with severe hydrops (*N* = 1), Skeletal dysplasia (*N* = 1), Cystic hygroma (*N* = 1), Spinal muscular atrophy with congenital fractures (*N* = 1), Congenital heart disease with single ventricle anatomy (*N* = 8), Thanatophoric dysplasia (*N* = 1), Glioblastoma (*N* = 1), Multiple malformations (*N* = 7), Trisomy 13 (*N* = 3). Outcomes included fetal demise (8), stillbirth (7) and live birth (33). The 33 newborns were treated with comfort care according to previously published guidelines^24^ and survival ranged from few hours to 127 days. Twenty-one parents submitted unsolicited written narrative commenting the experience of their newborns’ care.

**Fig. 1:**
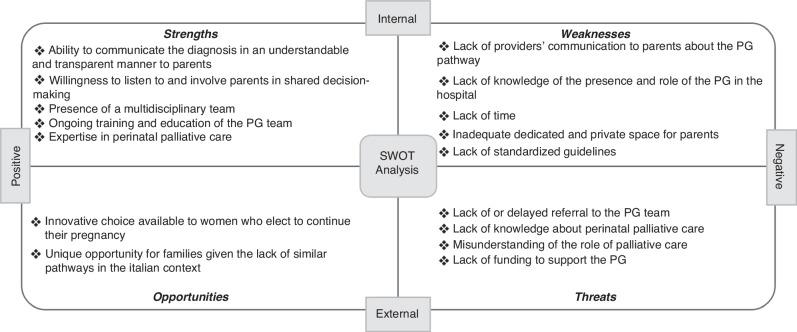
Summary of Strengths, Weaknesses, Opportunities, and Threats analysis of Giacomo’s Pathway.

Figure 1 shows the attributes summarized in SWOT categories. The strengths are characterized by the semantic field of the relationship (quote from a parent narrative: *“when I met your team, I finally felt relieved […]”*), while the weaknesses concern issues relating to organization and resources (quote from a PG team member: *“It was hard to dedicate plenty of time to this family along with our responsibilities in the NICU […]”)*. With regard to the opportunities, the innovation and the necessity of such a pathway emerged (quote from a parent narrative: *“For us, the Giacomo’s pathway was that light at the end of the tunnel, the hope that our little girl could also have the embrace of her mummy, her daddy and her brothers or whoever we wanted in our private moment”)*. Threats were characterized by lack of knowledge of the subject leading to difficult integration in everyday practice (quote from a PG team member “*It was challenging to involve the primary neonatologist in the palliative plan of care, I could feel her hesitation […]”)*.

Thirty-eight questionnaires were sent to 30 professionals and 8 parents, and 27 answers were collected and analyzed. Table 1 shows the results of evaluation through questionnaire. The responders included 8 physicians, 13 nurses and midwives and 6 parents.

**Table 1:** Average and standard deviation in descending order per category of the SWOT analysis.

	Attributes	Average	SD
Strengths	Ability to communicate the diagnosis in an understandable and transparent manner to parents	4.72	0.57
	Willingness to listen to and involve parents in shared decision-making	4.48	0.85
	Presence of a multi-disciplinary team	4.09	0.73
	Ongoing training and education of the PG team	4.04	0.71
	Expertise in perinatal palliative care	3.17	0.94
Weaknesses	Lack of providers’ communication to parents about the PG pathway	4.17	0.98
	Lack of knowledge of the presence and role of the PG in the hospital	4.09	1.00
	Lack of time	3.83	1.23
	Inadequate dedicated and private space for parents	3.83	1.37
	Lack of standardized guidelines	2.96	0.93
Opportunities	Innovative choice available to women who elect to continue their pregnancy	4.39	0.94
	Unique opportunity for families given the lack of similar pathways in the Italian context	3.70	0.97
Threats	Lack of or delayed referral to the PG team	4.39	0.94
	Lack of knowledge about perinatal palliative care	4.33	0.97
	Misunderstanding of the role of palliative care	4.26	0.96
	Lack of funding to support the PG	3.61	0.98

## Discussion

In this study we present features (strengths, weaknesses, opportunities, and threats) of a service of perinatal palliative care, the PG, by using SWOT analysis.

### Strengths

The main finding is that communication is a pivotal point of our program. The items *“Ability to communicate the diagnosis in an understandable and transparent manner to parents”* and *“Willingness to listen to and involve parents in shared decision-making”* were considered the most important strengths in this study. It is well recognized that the moment of the communication of a prenatal diagnosis is a delicate and critical step and determines the experience of the reminder of the pregnancy.^28,29^ When a woman is informed of a fetal LLC diagnosis the pregnancy turns, from a time of hope and expectation to a time of apprehension, anxiety, because of difficult decisions.^30^ Careful communication with the family is essential to provide all the necessary information, in a comprehensive and transparent manner to the family, facilitating decisions aligned with the family’s cultural beliefs and values. The communication method should be aimed at creating a relationship of trust between the team and the parents, essential for the safe and effective management of the entire process.

Other strengths of the study include *“Ongoing training and education”* and *“Expertise in perinatal palliative care*”. It is essential for professionals in perinatology to be proficient in PPC in order to be able to face the complexity of the medical and non-medical care of the dyad mother/baby. In fact, despite various recommendations for training in the field of perinatal palliation,^21,31^ gaps in knowledge and competence are still present.^32^ Thus, these results suggest the importance of developing training courses and offering formal education to professionals to facilitate competence in perinatal communication.^33,34^

Finally, the “*Presence of a multi-disciplinary team*” has been identified as a strength of the PG, mirroring what has been abundantly reported in literature.^26,30^

### Weaknesses

A timely prenatal palliative counseling is essential in the settings of a fetal diagnosis of LLC when the parents elect to continue their pregnancy. Parents need to have a good knowledge of the wide range of prognoses associated with a specific diagnosis in order to make an informed decision regarding delivery and postnatal plan of care. The earlier the perinatal journey starts, the easier is for parents to be ready for the celebration of the birth of their baby, especially when the infant’s life is expected to be short.^25,29,35^

The results of this study identified some weaknesses related to the process of prenatal counseling of the PG. The highest score went for *“Lack of providers’ communication to parents about the PG pathway”* and *“Lack of knowledge of the presence and role of the PG in the hospital”*. Narrative from families reported difficulties in coming to the knowledge of the option of PPC and of the presence of the program in our institution. *“It was not easy to find it* (the PG) *and realise that we too could be part of it”*. This aspect is certainly associated with the lack of understanding of providers about the role of palliative care,^20^ especially in the perinatal field. “*Lack of standardized guidelines”* obtained a relatively low score, reflecting the fact that the PG team developed its guidelines.^24^ However, “*Lack of time”* and *“Inadequate dedicated and private space for parents”* remain challenging issues, most likely associated with one of the threats: *“Lack of funding to support the PG”*. Team members make themselves available to the PG patients and their families in addition to their hospital responsibilities and the space in our institution is quite limited, thus, despite our best attempts, it has been difficult to assure privacy. These limitations have been mentioned by other authors.^36^

### Opportunities

Building a new PPC program requires motivation, time, energy and funds; however, it is clear that this type of care is needed and has been proven to be beneficial^12,29,37^ as an *“Innovative choice available to women who elect to continue their pregnancy”*. It is also crucial to point out that the PG constitutes a *“Unique opportunity for families given the lack of similar pathways in the Italian context”*. Unfortunately, PPC in Italy is not yet routinely integrated in the care of pregnancies with fetal LLC diagnosis, and only few centers provide a comprehensive service of PPC.^15^

### Threats

This study identifies several factors which threatened the success of the PG including the “*Lack of funding to support the PG”*, leading to some of the weaknesses identified above. Moreover, we believe that the *“Lack of knowledge about perinatal palliative care”* and the *“Misunderstanding of the role of palliative care”* led to the “L*ack of or delayed referral to the PG team”*, as reported by some families’ narrative. Fruitful attempts to educate perinatal professionals to the knowledge and the role of PPC and, specifically the service of the PG, have been the organization of annual conferences and training courses led by national and international experts in this field.^38^

In conclusion, the SWOT analysis of the 10-year experience of the PG showed important weaknesses and threats, mostly associated with lack of knowledge of the role of PPC, the presence of the PG in the institution, and lack of resources. However, the results demonstrated that the PG provided beneficial services to women faced with fetal LLC diagnoses. Communication and parents’ involvement in shared decision-making scored quite high in strengths. Moreover, the PG proved to be an innovative choice for women who elect to continue the pregnancy, and pretty unique in the Italian context, given the lack of similar programs. The narrative of one family sums it up: *“The Percorso Giacomo for us was the light at the end of the tunnel, the hope that our little girl could enjoy the embrace of her mum, her dad and her brothers and whoever we wanted in the precious and private moment of her birth”*.

## Data Availability

The datasets generated and analyzed during the current study are available upon request to the corresponding author.
